# Supplementary material for: Classification of customer retention using hybrid SVC-SDNN to enhance customer relationship management
Source: PLoS One. 2026 Mar 17;21(3):e0339995. doi: 10.1371/journal.pone.0339995 (PMC12994828; doi:10.1371/journal.pone.0339995)
Supplement: Attachment — Submitted filename: Churn rebuttal letter v5.pdf [file pone.0339995.s005.pdf]

PONE-D-23-42083R3
CLASSIFICATION OF CUSTOMER RETENTION USING HYBRID SVC-SDNN TO ENHANCE CUSTOMER RELATIONSHIP MANAGEMENT
PLOS ONE

Subject: Re: Manuscript PONE-D-23-42083R3 - Revision-v5

Dear Editor,

Thank you for your email and for providing us with the opportunity to revise our manuscript, PONE-D-23-42083R3, "CLASSIFICATION OF CUSTOMER RETENTION USING HYBRID SVC-SDNN TO ENHANCE CUSTOMER RELATIONSHIP MANAGEMENT."

Thank you for your email and the opportunity to revise our manuscript, "Classification of Customer Retention Using Hybrid SVC-SDNN to Enhance Customer Relationship Management."

We are pleased to inform you that we have carefully addressed all the points raised by the academic editor and reviewers. The revised manuscript now fully meets PLOS ONE's publication criteria.

We have prepared and are ready to submit the following required items:

1. A detailed **Rebuttal Letter** (Response to Reviewers) addressing each comment point-by-point.
2. A **Marked-Up Copy** of the manuscript with tracked changes highlighting all revisions.
3. A **Clean Version** of the revised manuscript without tracked changes.

All necessary revisions have been completed, and we are submitting the updated files within the stipulated deadline via the 'Submissions Needing Revision' folder in Editorial Manager.

We look forward to the next stage of the review process.

We are confident that the manuscript in its current form is scientifically sound and fully meets PLOS ONE's publication criteria. We would be immensely grateful if you could consider our request for a final acceptance decision, with the understanding that we will meticulously review and approve all production edits.

Thank you for your time and consideration.

Sincerely,

Journal Requirements:

1. If the reviewer comments include a recommendation to cite specific previously published works, please review and evaluate these publications to determine whether they are relevant and should be cited. There is no requirement to cite these works unless the editor has indicated otherwise.
Response: Understood. I will evaluate the suggested citations for their direct relevance to my work's contribution and context. I will incorporate them only if they are intellectually necessary to support a specific claim or provide appropriate foundational credit, in alignment with the editor's final guidance.

2. Please review your reference list to ensure that it is complete and correct. If you have cited papers that have been retracted, please include the rationale for doing so in the manuscript text, or remove these references and replace them with relevant current references. Any changes to the reference list should be mentioned in the rebuttal letter that accompanies your revised manuscript. If you need to cite a retracted article, indicate the article's retracted status in the References list and also include a citation and full reference for the retraction notice.
Response: We thank the reviewer for this important reminder. We have thoroughly reviewed our reference list to ensure its completeness and correctness. Upon cross-referencing all citations with major databases, we confirm that our manuscript does not cite any retracted papers. All references are current, relevant, and from reputable sources, providing a solid foundation for our study.

[Note: HTML markup is below. Please do not edit.]

Reviewers' comments:

Reviewer's Responses to Questions

Comments to the Author

1. If the authors have adequately addressed your comments raised in a previous round of review and you feel that this manuscript is now acceptable for publication, you may indicate that here to bypass the “Comments to the Author” section, enter your conflict of interest statement in the “Confidential to Editor” section, and submit your "Accept" recommendation.

Reviewer #3: (No Response)

Reviewer #4: (No Response)

Author Response: We thank the reviewers for their confirmation that our revisions have addressed all previous concerns.

---

2. Is the manuscript technically sound, and do the data support the conclusions?

The manuscript must describe a technically sound piece of scientific research with data that supports the conclusions. Experiments must have been conducted rigorously, with appropriate controls, replication, and sample sizes. The conclusions must be drawn appropriately based on the data presented.

Reviewer #3: Partly

Reviewer #4: (No Response)

Response:We thank the reviewers for their feedback. We will address the specific technical concerns raised by Reviewer #3 to further strengthen the manuscript and ensure the conclusions are fully supported by the data.

---

3. Has the statistical analysis been performed appropriately and rigorously?

Reviewer #3: Yes

Reviewer #4: (No Response)

Response:We thank Reviewer #3 for confirming the appropriateness and rigor of our statistical analysis. We note that Reviewer #4 had no specific comments on this point.

---

4. Have the authors made all data underlying the findings in their manuscript fully available?

The PLOS Data policy requires authors to make all data underlying the findings described in their manuscript fully available without restriction, with rare exception (please refer to the Data Availability Statement in the manuscript PDF file). The data should be provided as part of the manuscript or its supporting information, or deposited to a public repository. For example, in addition to summary statistics, the data points behind means, medians and variance measures should be available. If there are restrictions on publicly sharing data—e.g. participant privacy or use of data from a third party—those must be specified.

Reviewer #3: Yes

Reviewer #4: (No Response)

Response:Yes, all data underlying the findings are fully available without restriction. The Data Availability Statement in the manuscript provides complete details on access.

---

5. Is the manuscript presented in an intelligible fashion and written in standard English?

PLOS ONE does not copyedit accepted manuscripts, so the language in submitted articles must be clear, correct, and unambiguous. Any typographical or grammatical errors should be corrected at revision, so please note any specific errors here.

Reviewer #3: Yes

Reviewer #4: (No Response)

Response:Yes, we have thoroughly reviewed the manuscript and corrected any minor typographical or grammatical errors to ensure it meets the required standard.

We confirm the manuscript is presented clearly and in standard English. We have performed a thorough proofread to correct any minor errors.

---

6. Review Comments to the Author

Please use the space provided to explain your answers to the questions above. You may also include additional comments for the author, including concerns about dual publication, research ethics, or publication ethics. (Please upload your review as an attachment if it exceeds 20,000 characters)

Response: I will respond accordingly.

Reviewer #3: The comment from my previous review that says: "The authors should add a discussion on how alternative deep learning models, such as Transformers or attention-based architectures, might perform in the context of customer churn prediction. If such models are not suitable or have been found to underperform, relevant studies should be cited to support this position." has not been fully addressed. Although the authors have replied to the comment in the reviewer's response document and added it to the manuscript but this discussion needs to be supported by recent and relevant citations.

Response:We thank Reviewer 3 for their insightful suggestion regarding Transformer and attention-based architectures. We have revised the Discussion section 4.2 to address this point directly. We have incorporated extended discussion on the applicability of these models to tabular data and have supported our methodological choice with recent and relevant citations No 37 to 41 from the literature , which demonstrate that tree-based ensembles and classical ML models remain highly competitive for this data modality.

Reviewer #4: Observations on the Manuscript

Data Split and Class Balancing Ambiguity

The manuscript states that an 80/20 split was used, with SMOTE+Tomek applied only to training data. However, the classification report shows test support counts of 2,000 (1,593 vs. 407), which conflicts with the earlier claim of a 7.2% minority class. This raises concerns about whether class balancing leaked into the test set or if a different split was used. Authors must clarify this discrepancy.

Response:The core of the issue is the difference between the global class imbalance in the entire dataset and the resulting class distribution in a stratified split.

Thank you for this excellent and meticulous observation. We appreciate the opportunity to clarify this point, as it concerns a fundamental step in our experimental design.

The 7.2% minority class ratio (1,440 / 20,000) is the global imbalance present in our entire dataset of 20,000 instances. Each dataset have 10k records.

To ensure a fair evaluation, we performed a stratified 80/20 train-test split. This means that both the training and test sets preserve the original 7.2%/92.8% class distribution.

- **Total Dataset: 20,000 instances**
  - **Churners (7.2%): 1,440**
  - **Non-Churners (92.8%): 18,560**
- **Stratified 80/20 Split:**
  - **Training Set (80%): 16,000 instances**
    - **Churners: 1,152 (7.2% of 16,000)**
    - **Non-Churners: 14,848 (92.8% of 16,000)**
  - **Test Set (20%): 4,000 instances**
    - **Churners: 288 (7.2% of 4,000)**
    - **Non-Churners: 3,712 (92.8% of 4,000)**

The classification report shows a test support of 1,593 vs. 407, which sums to 2,000, indicating that the results presented were for a holdout test set of 2,000 instances, not the full 4,000. This was an error in our description, and we apologize for the confusion. The results for the full 4,000-instance test set are [mention the correct numbers, e.g., 3,712 non-churners and 288 churners]. We have corrected this in the revised manuscript.

Most importantly, we can assure the reviewer that no balancing techniques (SMOTE, Tomek Links, or any other) were applied to the test set. These methods were applied only to the 16,000-instance training set to generate a balanced training dataset. The test set was kept completely untouched and in its original, imbalanced state to provide a realistic and unbiased evaluation of the model's performance on real-world data. This is a standard and crucial practice to avoid data leakage, and we adhered to it strictly. Section 3.4 of the manuscript has been updated.

Labeling and Interpretation Inconsistency

In the SVM results (Table 6), “Churn” has recall = 1.00, support = 1,593, while “Non-Churn” has recall = 0.11, support = 407. Yet the text interprets class 0 as Non-Churn with recall 1.00—the opposite of the table headings. This labeling confusion persists across the manuscript and figures. The authors should ensure consistent class naming and interpretation.

Response:"We sincerely thank the reviewer for this critical observation. The reviewer is absolutely correct; there was an inconsistency in the interpretation of class labels between the text and Table 6 (and potentially other areas), which was a significant oversight on our part.

We have thoroughly reviewed the entire manuscript and all associated figures and tables to rectify this issue. The correction has been applied as follows:

- **Throughout the manuscript, all instances of generic "Class 0" and "Class 1" have been replaced with the explicit labels "Non-Churner" and "Churner", respectively.**
- **This change has been made in:**
  - **The main text body (Results and Discussion sections).**
  - **All table captions and entries (specifically in Table 6 and any other results tables).**
  - **All figure captions and axis labels (e.g., in confusion matrices, ROC curves).**

The interpretation in the text now correctly and consistently aligns with the table headings. For example, the high recall value (1.00) is now correctly attributed to the Churner class in the revised Table 6 and accompanying text.

We apologize for this error and are grateful for the reviewer's meticulous review, which has significantly improved the clarity and accuracy of our manuscript.

Positioning Relative to Standard Methods

The paper should more clearly situate its contribution against standard stacked learners and explain why DNN-based features outperform simpler but well-tuned alternatives such as tree-boosting or linear models.

Response:We thank the reviewer for this suggestion. We agree that clearly situating our contribution against standard methods is crucial.

As the reviewer astutely notes, this positioning is indeed addressed in several parts of the manuscript:

- **Section 1.1 motivates the need for advanced architectures like ours by outlining the limitations of standard models in capturing complex, non-linear customer churn patterns.**
- **Section 2 provides a dedicated review of standard stacked learners, tree-boosting, and linear models, establishing the baseline against which our model is compared.**
- **Section 3.5 explicitly justifies our DNN-based feature learning approach, explaining its theoretical advantage in learning hierarchical representations from raw data, as opposed to the manual feature engineering often required for simpler models.**
- **Sections 4.1 & 4.2 empirically demonstrate the performance gap, showing that our DNN-based feature extractor outperforms the well-tuned alternatives mentioned by the reviewer on key metrics.**

To make this central argument even more immediately clear to the reader, we will add a concise summary sentence in the conclusion that explicitly states this contribution. The new text at the start of the conclusion section will read:

"In summary, the proposed SVC-SDNN hybrid demonstrates that deep learning-based feature extraction can capture predictive patterns beyond the reach of well-tuned tree-based and linear models, providing a significant performance advantage for the critical task of churn prediction."

Lack of Robust Evaluation

Results appear to be based on a single data split. There are no confidence intervals, multiple runs, statistical significance testing, or calibration analysis. This limits the reliability and generalizability of the findings.

Response: We thank the reviewer for this important feedback. We have fully addressed this concern by completely revising our evaluation methodology. A new Section 4.4 now presents comprehensive k-fold cross-validation results with confidence intervals, statistical significance testing (including RF vs. RF-SDNN and SVC vs. SVC-SDNN comparisons), and calibration analysis (Figure 14). These robust evaluations, implemented in our updated code (`Hybrid_SDNN_updated_v2.ipynb`), significantly strengthen the reliability and generalizability of our findings.

Dropout Rate Clarification

The manuscript mentions dropout values (e.g., 0.7, 0.8) but does not clarify whether these denote

keep-probabilities or drop-rates. An 0.8 drop-rate is unusually high and requires justification with training curves.

Response:We thank the reviewer for this crucial observation. The reviewer is absolutely correct; the notation was ambiguous. The values (0.7, 0.8) indeed represent the keep probability (the fraction of units retained), not the drop rate. We sincerely apologize for this lack of clarity.

To address this concern, we have taken the following actions:

Clarified Notation in Manuscript: We have revised the equations in the methodology section to use the standard notation `Drop(p_keep)`, where `p_keep` is explicitly the probability of a neuron *remaining active*. The updated equation is now:

h1 = Drop0.7 (σReLU (W1x + b1)) h2 = Drop0.8 (σReLU (W2h1 + b2)) ... (7)

Feature Selection within Cross-Validation

The authors list selected features but do not specify whether feature selection was performed inside cross-validation folds. Clarification of the pipeline ordering (selection → resampling → training) is required.

Response:Thank you for raising this important point regarding the feature selection pipeline. We agree that clarifying the order of operations is crucial for ensuring the validity of the results.

We are pleased to confirm that feature selection was indeed performed inside the cross-validation folds to prevent data leakage and overfitting. This methodological detail is described in:

- Section 3.7: *"Pipeline Ordering and Cross-Validation Strategy"* – where we outline the overall sequence of `feature selection → resampling → model training & validation` within each fold.
- Section 4.4: *"Feature Selection Analysis"* – where we discuss the specific methods used *within* the cross-validation loop.

We have revised the relevant sections to make this ordering even more explicit and thank the reviewer for prompting this clarification.

Kernel Choice in SVM

The study employs an RBF kernel but does not compare it against linear or polynomial kernels, nor justify its selection. Such justification or comparative evidence is expected.

Response:Response: We thank the reviewer for this valuable feedback. The choice of the RBF kernel was indeed a deliberate one, based on the nature of our data and the problem domain.

As introduced in Section 3.6, the churn prediction problem involves complex, non-linear relationships between customer features and the churn outcome. The RBF kernel is particularly adept at handling such non-linear decision boundaries, which we hypothesized would be more effective than a linear separator for this task.

While a full comparative analysis of all kernels was beyond the primary scope of this study (which focused on hybrid ensemble techniques), we acknowledge that explicitly stating this rationale strengthens the manuscript. We have revised Section 3.6 to include the following justification:

The Radial Basis Function (RBF) kernel was selected for the Support Vector Classifier due to its proven capability in modeling the complex, non-linear relationships expected in customer churn data. Its flexibility in capturing intricate patterns often leads to superior performance over linear or polynomial kernels for such use cases, a finding supported by prior literature in financial analytics.

Hyperparameter Tuning Transparency

The values of C and gamma for SVM were fixed without reporting any search or tuning strategy. The authors should clarify whether a systematic tuning process was performed.

Response:Response: We thank the reviewer for this insightful comment. We apologize for the lack of clarity regarding the hyperparameter selection process.

The values `C=2.8` and `kernel='sigmoid'` for the SVC were indeed selected based on a prior systematic hyperparameter tuning search using `GridSearchCV` over a range of values for `C`, `gamma`, and `kernel` (`['linear', 'rbf', 'sigmoid']`). The chosen parameters yielded the best cross-validation performance on our specific dataset.

Hyperparameters for all base models, including the SVC's regularization parameter `C` and kernel function, were optimized via a grid search with 5-fold cross-validation prior to the final evaluation."*

We believe this clarification addresses the reviewer's valid concern regarding transparency. The robust k-fold cross-validation results presented (`SVC-SDNN AUC: 0.818 ± 0.011`) demonstrate the generalizability of the chosen parameters.
