# Supplementary material for: Classification of customer retention using hybrid SVC-SDNN to enhance customer relationship management
Source: PLoS One. 2026 Mar 17;21(3):e0339995. doi: 10.1371/journal.pone.0339995 (PMC12994828; doi:10.1371/journal.pone.0339995)
Supplement: Attachment — Submitted filename: Churn rebuttal letter v6.pdf [file pone.0339995.s006.pdf]

Subject: Rebuttal Letter – Manuscript PONE-D-23-42083R4 (Revision 6)

Title: *Classification of Customer Retention Using Hybrid SVC-SDNN to Enhance Customer Relationship Management*

Dear Editorial Office and Reviewers,

We thank you and the reviewers for the careful evaluation of our manuscript and for the constructive feedback that has helped us improve the presentation of our work. Below we address each comment point-by-point. As the remaining issues are minor typographical or formatting matters, we note that they will be fully resolved during the proof-reading and production stage, in accordance with the journal's standard workflow.

Reviewer #3

1. **Reference to the reviewer in the main text**

   Comment: The phrase "the reviewer rightly points out…" is inappropriate for the manuscript.

   Response: We agree. The sentence has been rewritten in an impersonal style:

   "Alternative deep-learning architectures such as Transformers and other attention-based mechanisms have achieved state-of-the-art results in domains with complex, high-dimensional data (e.g., natural-language processing and computer vision)."

   Incomplete citation placeholders ("[?]")

   Comment: Several citations appear as placeholders, likely due to a LaTeX formatting issue.

   Response: The missing reference keys have been corrected, and the bibliography now

compiles without errors. All citations are properly rendered in the final PDF.

Reviewer #4

1. **Table of hyper-parameters and their tuning**

   Comment: Add a clear table summarizing hyper-parameters.

   Response: A new Table 2 (Hyper-parameter Configuration) has been inserted into the Methods section, listing each parameter, its search range, and the selected value after tuning.

   Experimentation environment (hardware & software)

   Comment: Specify the computational resources and software stack used.

   Response: We have added a paragraph at the end with the title "Experimentation parameters". And text 'All experiments were conducted on a system equipped with an Intel Core i7-12700K processor, 12GB of DDR4 RAM, and a colab or kaggle based GPU and TPU with at least 12 GB of VRAM. The use of a GPU or TPU is essential for accelerating the training process of the Stacked Deep Neural Network (SDNN) component of our hybrid model.'

General Remarks

All remaining minor issues—such as line-break inconsistencies, figure caption refinements, and typographic polishing—have been noted and will be addressed during the proof-reading stage prior to final publication. We trust that these revisions satisfy the reviewers' concerns and bring the manuscript to the high standard expected by *PLOS ONE*.

We appreciate the opportunity to improve our work and look forward to the next steps in the editorial process.

Respectfully,

Authors
