# Supplementary material for: Classification of customer retention using hybrid SVC-SDNN to enhance customer relationship management
Source: PLoS One. 2026 Mar 17;21(3):e0339995. doi: 10.1371/journal.pone.0339995 (PMC12994828; doi:10.1371/journal.pone.0339995)
Supplement: Attachment — Submitted filename: PLOS ONE - Appeal Request Form .docx [file pone.0339995.s002.docx]

PLOS ONE Appeal Request Form

Please fill out the below form with the relevant information. We require a response to all three sections before we can evaluate your appeal request.

# 1. Academic Editor

Our standard process is to consult Academic Editors on appeals on decisions they handled. If you have any concerns that a consultation with the Academic Editor may unduly affect the evaluation of your appeal, please indicate so along with a brief summary of the reasons for your concerns.

☐ I do not have any concerns about the Academic Editor being consulted on this appeal.

☒ I do have concerns that the Academic Editor being consulted may unduly affect the evaluation of my appeal for the following reasons:

**Comments 1**:

After careful consideration, we have decided that your manuscript does not meet our criteria for publication and must therefore be rejected.

**Response 1**:

I was busy reviewing your already-published relevant manuscripts on Google Scholar.

Before Submission and after receiving an email regarding the decision, I thoroughly reviewed the recent relevant articles in PLOS ONE.

The description of the models is not schematic or superficial. Most of the important concepts are mentioned on a need-by-need basis (as required).

Many of PLOS ONE's and your recent articles follow the same structured approach.

**Comment 2**:

I am sorry that we cannot be more positive on this occasion, but hope that you appreciate the reasons for this decision.

**Response 2:**

There is a comprehensive presentation of the problem and its peculiarities. Could you please elaborate on the in-depth description of your rejection decision?

**Comments 3:**

This paper does not provide a solid method for Accuracy and AUC-ROC of SVC-SDN.

**Response 3:**

The method for Accuracy and AUC-ROC of SVC-SDN is exactly the same as we find it in recent relevant literature. The problem is also characterized, and the evaluation of each approach is given with results.

**Comment 4:**

In addition, the paper structure is not well managed.

**Response 4:**

PLOSONE initially allow articles submission in open format. After acceptance we are bound to fully adopt the Journal standard structure.

# 2. Editorial Policies

If you have concerns that the decision deviates from one of our editorial policies, please provide us with some brief notes on the specific policy.

☐ I do not have any concerns about this decision deviating from PLOS ONE’s editorial policies.

☒ I do have concerns that this decision deviated from PLOS ONE’s editorial policies for the following reasons:

**Comment 5:**

Also, there is no comparison in the related work section.

**Response 5:**

SVC-SDN is compared with several other recent approaches in the related work as well as results and experimental section.

**Comments 6:**

The references are quite old.

**Response 6:**

What can we do with old references of scientific importance? In my opinion we must include it. There are plenty of recent references.

I even shared the notebook on github and the churn dataset links.

There are more recent variants in the latest press, and it will take time to work them out as a future work.

I personally find many typos and grammar mistakes in published articles and even in your Decision Email.

The Sign of a true researcher is a rational and objective mindset. Many Editors/Reviewers follow **prejudices.**

**Comments 7:**

I am sorry that we cannot be more positive on this occasion, but hope that you appreciate the reasons for this decision.

**Response 7:**

Based upon the above point-by-point response of your editorial opinion, we are quite hopeful to reconsider the same article for onward processing.

**Comments 8:**

The accuracy claim is too high but its not well explained.

**Response 8:**

As a programmer Its really time consuming and tedious task to improve the model performance over a dataset. Several layers are added and removed for better results.

Layer in any deep NN represent either a neuron or a network of motor, Sensory and associative neurons.

# 3. Response to Reviewers: **Not reviewed yet N.A**

# Please provide a point-by-point response to the reviewers and any Academic Editor’s comments in a separate document.

☐ I confirm that I have attached a Response to Reviewers as a separate file to this email in addition to this form.

4. Track Changes Manuscript: No changes made N.A

If you made any updates to your manuscript, please also provide a ‘Track Changes’ version of your manuscript that shows any changes you may have made to your submission.

☐ I confirm that I have attached a ‘Track Changes’ version of my MS as a separate file to this email in addition to this form and Response to Reviewers.
