# Supplementary material for: Classification of customer retention using hybrid SVC-SDNN to enhance customer relationship management
Source: PLoS One. 2026 Mar 17;21(3):e0339995. doi: 10.1371/journal.pone.0339995 (PMC12994828; doi:10.1371/journal.pone.0339995)
Supplement: Attachment — Submitted filename: response to reviewers.docx [file pone.0339995.s003.docx]

Dear Editor and reviewers, we are really thankful for your valuable suggestion and comments. Accordingly, we made needful corrections to further improve this manuscript. We rectified/amend the article as directed in the instructions and comments raised by reviewers panel. Comprehensive response and corrections are listed in the table below.

| PONE-D-23-42083R1 |  |
| --- | --- |
| Journal requirements: | Author Response and corrections |
| 1. Please ensure that your manuscript meets PLOS ONE's style requirements, including those for file naming. The PLOS ONE style templates can be found at <https://journals.plos.org/plosone/s/file?id=wjVg/PLOSOne_formatting_sample_main_body.pdf> and <https://journals.plos.org/plosone/s/file?id=ba62/PLOSOne_formatting_sample_title_authors_affiliations.pdf> | We adopted the standard PLOS ONE latex template. Additional packages were used to further improve the article overall structure.  We have to share/upload the following documents   1. Manuscript in PDF 2. Highlighted text copy of the Manuscript in PDF 3. Latext file 4. Cover Letter. |
| 2.We suggest you thoroughly copyedit your manuscript for language usage, spelling, and grammar. If you do not know anyone who can help you do this, you may wish to consider employing a professional scientific editing service. Whilst you may use any professional scientific editing service of your choice, PLOS has partnered with both American Journal Experts (AJE) and Edit age to provide discounted services to PLOS authors.  Upon resubmission, please provide the following: ● The name of the colleague or the details of the professional service that edited your manuscript ● A copy of your manuscript showing your changes by either highlighting them or using track changes (uploaded as a supporting information file) ● A clean copy of the edited manuscript (uploaded as the new manuscript file) | The First author is a paid reviewer of AJE and already used to the level and quality of AJE standard contents. The article text is revised and verified by many other relevant experts. We are also used to AI enabled tools and techniques.  We enhance the language and grammar of this Manuscript with extra ordinary efforts. The article already undergone comparative analysis with existing online PLOS ONE recent published work.  A detail acknowledgement is rewritten. Upon Resubmission, a manuscript copy with highlighted text and another clean copy will be uploaded. |
| 3. When completing the data availability statement of the submission form, you indicated that you will make your data available on acceptance. We strongly recommend all authors decide on a data sharing plan before acceptance, as the process can be lengthy and hold up publication timelines. Please note that, though access restrictions are acceptable now, your entire data will need to be made freely accessible if your manuscript is accepted for publication. This policy applies to all data except where public deposition would breach compliance with the protocol approved by your research ethics board. If you are unable to adhere to our open data policy, please kindly revise your statement to explain your reasoning and we will seek the editor's input on an exemption. Please be assured that, once you have provided your new statement, the assessment of your exemption will not hold up the peer review process. | A data availability statement is added in the Manuscript. The Statement consist of a proper weblink of the coding file namely ‘SVC-SDN-Churn.ipnyb’ and the publicly available dataset namely’ customer-churn-data.csv’. |
| Additional Editor Comments (if provided): | No Comment. |
| Reviewers' comments: Reviewer's Responses to Questions **Comments to the Author** |  |
| 1. If the authors have adequately addressed your comments raised in a previous round of review and you feel that this manuscript is now acceptable for publication, you may indicate that here to bypass the “Comments to the Author” section, enter your conflict of interest statement in the “Confidential to Editor” section, and submit your "Accept" recommendation.  Reviewer #1: (No Response)  Reviewer #2: (No Response) | Dear Editor,  As we submit our resubmission, it appears both reviewer remains convinced, despite revisions that have satisfied all others. Given the substantial progress and positive feedback from the majority of reviewers, we kindly request that you consider making a direct editorial decision on our paper at this juncture.  Thank you for your consideration.  Best regards  Authors |
| . Is the manuscript technically sound, and do the data support the conclusions?  The manuscript must describe a technically sound piece of scientific research with data that supports the conclusions. Experiments must have been conducted rigorously, with appropriate controls, replication, and sample sizes. The conclusions must be drawn appropriately based on the data presented.  Reviewer #1: Yes  Reviewer #2: Yes | The author Naila Yaqub is currently working in Khyber bank. She motivated us to address these real-world issues for banker community. Especially the new commers face embarrassment and even job loss due to high churn ratio. |
| 3. Has the statistical analysis been performed appropriately and rigorously?  Reviewer #1: N/A  Reviewer #2: Yes | Change in layer structure and the number of dropout layer may further improve the precision of prediction. |
| 4. Have the authors made all data underlying the findings in their manuscript fully available?  The [PLOS Data policy](http://track.editorialmanager.com/CL0/http:%2F%2Fwww.plosone.org%2Fstatic%2Fpolicies.action%23sharing/1/010f019524e975e7-b32e4d3c-5be3-4bb4-b055-75c6de03ca2b-000000/CjqDtXbtMV2D0agwHcGy5rOPkshDUx6Sj7kMLIbu19I=199) requires authors to make all data underlying the findings described in their manuscript fully available without restriction, with rare exception (please refer to the Data Availability Statement in the manuscript PDF file). The data should be provided as part of the manuscript or its supporting information, or deposited to a public repository. For example, in addition to summary statistics, the data points behind means, medians and variance measures should be available. If there are restrictions on publicly sharing data—e.g. participant privacy or use of data from a third party—those must be specified.  Reviewer #1: Yes  Reviewer #2: Yes | The Data availability statement is added. Coding and dataset are fully available without restrictions. |
| 5. Is the manuscript presented in an intelligible fashion and written in standard English?  PLOS ONE does not copyedit accepted manuscripts, so the language in submitted articles must be clear, correct, and unambiguous. Any typographical or grammatical errors should be corrected at revision, so please note any specific errors here.  Reviewer #1: (No Response)  Reviewer #2: Yes | The article is amended and improved according to the valuable comments of reviewers.  As an AJE paid reviewer all the important aspects of a standard article is fulfilled. |
| 6. Review Comments to the Author  Please use the space provided to explain your answers to the questions above. You may also include additional comments for the author, including concerns about dual publication, research ethics, or publication ethics. (Please upload your review as an attachment if it exceeds 20,000 characters) | We will do it accordingly. |
| Reviewer #1: The paper presents improved churn detection using novel hybrid deep learning algorithms to predict bank customers’ retention and identify factors that influence better customer retention. SVC-SDNN model was proposed for churn prediction. The proposed model was compared to other existing models with the proposed model having a better performance. The performance of the proposed model is encouraging. However, I have the following observations: 1. The claim in second paragraph under section 1.1 should be cited | Relevant citation is added in the revised article. |
| 2. Sections 1.2 to 1.5 are not needed in a paper. I think those sections are relevant to project/thesis report. If there is anything important at all in those sections, especially, section 1.5 that talks about the structure of the paper should be married to the introduction as the last paragraph. | We are really tankful to your valuable suggestions. All changes have been incorporated. Unnecessary text is deleted and the article organization section is merged with introduction. |
| Figure 1 citation is incomplete, it should be a surname, year. | The citation is done. PLOS ONE latext template encounter error in using the cite {} command. |
| Section II should be renamed as Background of the Study and Related Work because Sections 2.1 and 2.2 fall under background | The changes have been incorporated and the section is renamed. |
| In-text citations in Table 1 do not need initials but only surname(s) and year. In addition, the table should provide a succinct summary of the related work and research gaps and not just a repetition of titles and dataset. | All changes were incorporated. The text summarizes the nature of work. |
| Remove any line of codes in the paper. Codes can be added as appendix or supplementary if required. | Removed and the coding file weblink is shared in the data availability statement. |
| The dataset is skewed as the authors have also notified. There is need to balance the dataset to avoid model over/underfitting. | Dataset in the below weblink is working fine with the given model layers and dropout layers for overfitting. <https://www.kaggle.com/datasets/bhuviranga/customer-churn-data>.  The layer structure of sequential DNN is evident in the below given notebook.  https://github.com/ishaqafridi/SVC-SDN-Churn |
| The authors claimed that relevant features were selected. However, what informed the selection of the relevant features or better still the technique for feature selection was not stated. | This sentence is added before the proceeding of the relevant work table in the recent literature section.  In the given dataset age, credit, balance and transaction information were usually used for churn prediction. |
| The hybridization of the SVC and SDNN was not clear in the paper. Authors are to provide the details of hybridization to enable reproducibility. | The hybridization is combined in the following code segment  #Prediction of SVM is hybridized with SDNN  y_pred_svmann = (y_pred_svm +  y_pred_ann.flatten()) / 2  #set new values for the 'SVC-SDNN'column  y_pred_svmann_ =  np.where(y_pred_svmann > 0.5, 1, 0)  np.save('y_pred_svmann_.npy',  red_svmann_) |
| Allow a line spacing before and after a figure/table for readability. | This is issues is resolved throughout the document. |
| I suggest you merge Tables 2 to 11 as one so that one can easily see the difference in performance among the models at a glance. | For more clarity each test results need to be displayed in a separate table. |
| The confusion matrices from Figures 9 to 13 can be arranged together or sequentially (may be 3 in a row) for readability and comparison. | Fixed and two matrices can be clearly arranged through \\begin{minipage}[b]{0.3\textwidth} latex code. |
| The structure of the paper is not good enough. It is advisable to follow the journal template. | The article is now in PLOS ONE approved latex template. |
| Reviewer #2: Although, I haven't previously review the paper, However, evaluating the authors response to the reviewers, authors have comprehensively addressed the reviewer comments in the revised version. Still, to improve the quality of the paper, below are certain comments that need to be address. | We are really thankful to your encouraging remarks. We are committed to excellence and we will do as directed. |
| The authors should add more technical details about the proposed approach in the abstract, i.e., talking more about preprocessing, feature engineering, or model details. | Technical details are added and the abstract has a new look. |
| The authors should enlist the key contribution of the proposed approach. | A specific SVC variant is used for classification. Sequential deep neural network layered structure has been adjusted so many times to improve the model performance. Introduction highlight the significance and motivation toward this real-world issue. SVC-SDNN subsection enlist the Novelty and timeliness of the proposed model. |
| The support vector machine and random forest should be moved to the result or methodology section where the proposed approach is elaborated, the related work should only focus on the state-of-the-art literature. | Respected Reviewer,  We have to discuss only relevant state-of-the-art literature.  SVM, DNN and the rest of algorithms were used for comparative evaluation.  There is only need based elaboration and explanation in respective sections. |
| Figure 5 seems unnecessary, instead a Table should be added that shows several example from the dataset. | It is visible in the shared coding notebook in original form. This is the screenshot of output table. The tabular figure shows all the attributes with non-null counts and their respective datatypes. |
| There are many deep-learning based approaches published on the topic. What authors motivate to proposed a hybrid approach while not using state-of-the-art transformers or LLMs? Adding a more rationale will further improve the justification of the proposed hybrid approach | Deep Neural networks perform convolution operation and are purely discriminative in nature.  The ability and suitability of each DNN with respect to a dataset is important. Large language models or transformers can be categorized as the generative type of AI.  For the mentioned Churn prediction dataset with the given attributes as shown in the figure 5, an improved hybrid sequential DNN is the best choice. |
| The authors should further provide details on the implementation of the proposed approach, such as on how SVM is added to the DNN classifier? | The hybridization is combined in the following code segment  #Prediction of SVM is hybridized with SDNN in this way.  y_pred_svmann = (y_pred_svm +  y_pred_ann.flatten()) / 2  #set new values for the 'SVC-SDNN'column  y_pred_svmann_ =  np.where(y_pred_svmann > 0.5, 1, 0)  np.save('y_pred_svmann_.npy',  red_svmann_) |
| The author should demonstrate or provide the rationale on how the simple banking system dataset attributes are enough for churn prediction for the generalizability of the proposed approach. | There are some important attributes or indicators associated with each location customers. The selection of attributes used in a dataset influence the correct prediction of churn. In our case age, balance, credit cards and credit score are very important in the precise prediction of churns. |
| Also, what motivate the authors for the proposed approach, as there are better alternatives to the proposed approach by employing large language models. | As earlier explained each AI algorithm is situation/issue aware. It mean that for every possible real world problem there are several optimized solutions. The performance of some methods for a certain kind of solution and some specific datasets is impressive. Churn prediction can be done with discriminative AI. Large Language models or generative AI is effective in some other application areas. |
| In Tables, the labels 0 and 1 should be replaced with more intuitive one to better understand the results. | The use of 0 and 1 is already explained below table 3. 0 is used for non-churner and 1 is used for churner customers.  It is exactly according to the general classes Nomenclature in AI programming. |
| Summary should be replaced with Discussion and add some insights how the proposed approach help state-of-the-art. | The title is changed and the following text is added “The performance of any AI discriminative deep neural network is influenced by the number and nature of attributes used by loyal customers.” |
| The authors should add threats to validity section to identify the possible threats in the proposed approach. | As per the directive of reviewer the validation sub section with the following text is added in the Manuscript after the proposed discussion section. “Validation of the proposed method  A serious threat to managerial decision process. Churn adversely affect Customer relationship management. The marketing campaign toward a large number of churn customers is a waste of time and resources. Efficient and effective smart AI solutions improve management decisions and investment.” |
