# Supplementary material for: Classification of customer retention using hybrid SVC-SDNN to enhance customer relationship management
Source: PLoS One. 2026 Mar 17;21(3):e0339995. doi: 10.1371/journal.pone.0339995 (PMC12994828; doi:10.1371/journal.pone.0339995)
Supplement: Attachment — Submitted filename: churn response to reviewers v4.docx [file pone.0339995.s004.docx]

Dear Editor and Reviewers,

We sincerely appreciate your time and the thoughtful feedback provided on our manuscript. While addressing your suggestions required significant effort and careful revision, we are grateful for the opportunity to improve our work.

### **Subject: Response to Customer_ID Anonymization Query.**

1. Clarification on Anonymization Status:
The customer_id fields in the provided 'Data' files are not direct or reversible representations of actual customer account numbers. They are internally generated identifiers used solely for data linkage within the licensed dataset.

2. Privacy Safeguards:

- No PII Exposure: The identifiers do not expose personally identifiable information (PII) or financial details (e.g., real account numbers, names, or contact information).
- Non-Reversible: The values cannot be traced back to individual customers without access to additional, restricted mapping tables (which are not part of the shared files).

3. License Compliance (DbCL v1.0):

- The dataset is governed by the Database Contents License (DbCL) v1.0, which permits redistribution of anonymized/non-personal data.
- The customer_id field adheres to this license as it does not contain raw PII. Removal is unnecessary unless explicitly required by the license’s terms or a regulatory mandate.

Regards

Authors
